# An Integrated Pest Management Strategy Approach for the Management of the Stable Fly *Stomoxys calcitrans* (Diptera: Muscidae)

**DOI:** 10.3390/insects15040222

**Published:** 2024-03-25

**Authors:** Mikel A. González, Gérard Duvallet, Damien Morel, Ignacio de Blas, Elena Barrio, Ignacio Ruiz-Arrondo

**Affiliations:** 1 Departamento de Biología de la Conservación y Cambio Global, Estación Biológica de Doñana (EBD-CSIC), 41092 Sevilla, Spain; mikel_alexander86@hotmail.com; 2 CIBER de Epidemiología y Salud Pública (CIBER ESP), 28029 Madrid, Spain; 3 Centre d’Écologie Fonctionnelle et Évolutive, Université Montpellier, CNRS, EPHE, IRD, Université Paul Valéry Montpellier 3, 34199 Montpellier, France; 4 Bestico, 44860 Pont St Martin, France; dmorel@bestico.fr; 5 Department of Animal Pathology, Faculty of Veterinary Sciences, Instituto Universitario de Investigación Mixto Agroalimentario de Aragón (IA2), Universidad de Zaragoza, 50013 Zaragoza, Spain; deblas@unizar.esp (I.d.B.); or irarrondo@riojasalud.es(I.R.-A.); 6 El Refugio del Burrito, 06394 Bodonal de la Sierra, Spain; elena.barrio@elrefugiodelburrito.com; 7 Center for Rickettsiosis and Arthropod-Borne Diseases, San Pedro University Hospital-CIBIR, 26006 Logroño, Spain

**Keywords:** *Stomoxys*, biological control agents, mass trapping, animal welfare, Donkey Sanctuary, *Macrocheles robustulus*, *Spalangia cameroni*, *Muscidifurax raptor*, Spain

## Abstract

**Simple Summary:**

Stable flies, known for inflicting painful bites on livestock, cause substantial economic losses worldwide by hindering animal feeding and potentially transmitting diseases. Current chemical control methods face challenges of resistance and environmental risks. To address this, an integrated pest management approach is proposed and tested in a sanctuary in western Spain. This strategy combines cultural practices, animal protection, the release of natural enemies targeting immature stages (predatory mites and two species of wasp parasitoids), and a specialized trapping system for adults. The study highlights the use of this holistic strategy toward a sustainable and environmentally friendly method for stable fly control. This approach aims to mitigate economic losses and reduce reliance on traditional insecticides, contributing to improved livestock well-being and ecosystem health.

**Abstract:**

Stable flies, *Stomoxys calcitrans*, stand as formidable pests with a global impact, inflicting significant economic losses on the livestock sector. Larval development occurs in diverse substrates, including decomposing plant material and manure, while emerged adults pose a threat through blood-feeding on both animals and humans. Conventional chemical control methods, predominantly reliant on insecticides, not only pose environmental risks but also face challenges of resistance among stable fly populations. To address this pressing issue, we propose an integrated pest management (IPM) strategy for stable fly control. This approach involved a combination of sanitary-cultural practices, animal protection, the release of natural enemies targeting immature stages, and a specialized trapping system for adults. The Stomoxycc^®^ trap, designed for mass trapping of adult *Stomoxys*, was employed alongside the release of the predatory mite *Macrocheles robustulus* and two wasp parasitoids, *Spalangia cameroni* and *Muscidifurax raptor* (under the commercial brands Biomite^®^ and Biowasp^®^) on animal bedding as a key component of this IPM strategy. The implementation of this initiative has been undertaken at a significant sanctuary for donkeys and mules in western Spain. In this publication, we present the application and results of the IPM strategy utilized and provide insights into its use as a sustainable and environmentally friendly option for controlling stable fly populations.

## 1. Introduction

Stable flies, scientifically known as *Stomoxys calcitrans*, pose a significant threat to livestock worldwide, causing substantial economic repercussions. In the US alone, they impose a significant financial burden, estimated at approximately between $840 million and $2300 million annually [1]. This estimation encompasses various expenses related to reduced productivity, veterinary expenses, and decreased animal welfare. Moreover, the detrimental effects extend beyond the US, reverberating in European countries. France experiences a staggering €145 million and €234 million in annual losses in the meat and milk sector, respectively, attributed to stable flies [2].

These blood-feeding insects are commonly found in livestock facilities and exhibit a biting behavior that poses severe discomfort and pain to animals. Such bites can lead to distress among animals, hindering their feeding behavior and resulting in reduced weight gain, milk production, and overall productivity. Additionally, the bites create open wounds that may serve as potential entry points for pathogens, increasing the risk of disease transmission among livestock [3,4,5]. The indirect effect of *S. calcitrans* on hosts is the mechanical transmission of pathogens, which in Europe is most likely limited to a narrow range of pathogens such as *Trypanosoma evansi* and *Besnoitia besnoiti* primarily, and potentially other exotic diseases, including Lumpy Skin Disease and Equine Infectious Anemia [4,6].

*Stomoxys* flies exert a particularly detrimental impact on equine and ruminant husbandry, significantly affecting the health, productivity, and well-being of these animals. Their preference for specific breeding substrates, such as donkey dung, contributes to their pervasive presence in livestock environments, intensifying the challenges faced by animal caretakers and farmers. Some studies highlighted the affinity of *Stomoxys* flies for donkey dung as a favored breeding site, emphasizing the implications of this behavior on the proliferation of these pests within livestock facilities and reinforcing the notion that these flies exhibit a distinct preference for donkey dung, further exacerbating their presence in equine settings and affecting ruminant husbandry practices [7,8,9].

These flies often prompt defensive behaviors in animals, such as tail swishing and foot stamping, to ward off biting pests [10]. Various avoidance behaviors have been explored in both livestock [11,12,13] and horses [10,14,15]. These behaviors, observed over specific timeframes, serve as proxy measures for the stable flynuisance, allowing comparisons across temporal observations to assess changes throughout a season. The impact of stable flies on livestock health and productivity underscores the urgency for effective pest management strategies to mitigate these substantial economic and animal welfare losses. However, traditional control methods, such as insecticides and repellents, are costly and often limited in efficacy [6,7].

Insecticides have been used commonly to control stable flies, but this approach comes with environmental damage and the challenge of resistance [16,17,18]. The resistance of flies to insecticides has been observed both phenotypically and genetically, necessitating alternative and sustainable control strategies. A promising alternative to chemical control is the use of parasitic wasps, including species like *Spalangia* spp. and *Muscidifurax* spp. (Hymenoptera, Pteromalidae). These natural enemies have demonstrated significant success in reducing stable fly populations in field trials by laying their eggs inside stable fly pupae, leading to the demise of the developing fly pupae [19]. Unlike chemical methods, the use of parasitic wasps is environmentally friendly and does not harm non-target organisms, offering a sustainable approach to stable fly control [6,7]. These beneficial insects are already integrated into global pest management systems [7]. In addition to parasitic wasps, predatory acari like *Macrocheles robustulus* (Berlese, 1904) (Acari, Macrochelidae) have shown their efficiency in the control of eggs and larvae. The predation capacity of Macrochelidae acari on fly eggs and newly hatched larvae ranges from 5 up to 15 hosts per day per predatory mite, depending on the host and predator species [20]. In addition to biological controls, another common practice to combat stable flies is the use of trapping systems, which can serve as surveillance–monitoring traps that can both catch and remove the population of stable flies. Trapping is a common practice extensively used as a primary barrier to combat these flies, and different attractiveness and efficacy are reported depending on trap models [6,7]. Thus, the search for the ideal trap is still an unresolved task that deserves further research.

The proposed integrated pest management strategy involves a combination of natural enemies targeting immature stages, a specific trapping system for adults, along with cultural and sanitary practices. This approach not only ensures effective control but also provides a safe and environmentally friendly option for stakeholders. By employing biological control agents and traps, the integrated pest management program outlined in this study aligns with sustainability goals, ease of use, and the overall well-being of both the environment and farmers. Additionally, it seeks to provide recommendations for improving operational protocols and data collection, with the goal of recommending the use of parasitic flies and predator acari.

## 2. Materials and Methods

### 2.1. Study Area

**Figure 1:**
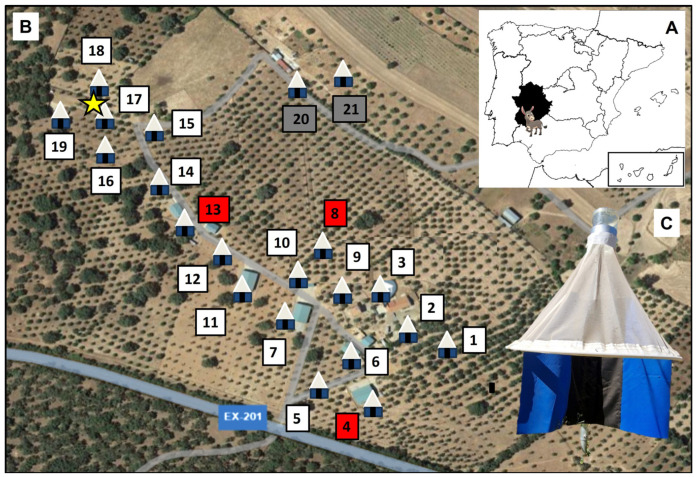
(**A**) Map of Spain showing the study region in black color. (**B**) Detailed picture of The Donkey Sanctuary showing the position of the 21 traps deployed (control traps: grey tones, selected monitoring traps: red tones, and massive trapping traps: ordinary color). Yellow star showing the pile of manure. (**C**) Detail of the Stomoxycc^®^ trap model installed in the sanctuary.

The study was conducted in The Donkey Sanctuary “El Refugio del Burrito”, on the Doña Rosa farm, which is in Bodonal de la Sierra, Badajoz province, western Spain, at 38° 9′ 25, 44′ N and 6° 34′ 32, 34′ W (Figure 1A). The farm covers an area of ca. 32 ha of pasture with ca. 2000 olive trees and 120 holm oaks (Figure 1B). The farm is located at an altitude of 610 m.a.s.l. It is situated in the catching area of the Guadiana River, having more proximity (approximately 68 km away) to the river Ardila. The climate is subhumid (600–1000 mm), with an average annual rainfall in 2022 of 1.07 and 0.73 L/m^2^ in 2023. The rainfall is very variable during the year, with a marked dry summer season. The rainiest months are October and December. The average sunshine rate for 2022 was 8.5 h in 2022 and 9.4 h in 2023. Climatic data on minimum and maximum temperature, precipitation, and daily hours of sunshine for the period 2021–2023 were obtained from the closest meteorological station in Jerez de Los Caballeros, located at 19 km longitudinal distance from the farm (AEMET).

### 2.2. Animal and Farm Characteristics

There are a total of 141 animals of mixed breeds at the farm, comprising 110 donkeys, 28 mules, and 3 horses, which are divided into seven barns depending on their needs. Each barn hosts between 2 to 23 animals. The main barns are constructed of a metal frame with internal and external concrete walls. On the side walls of the barns, above, there are plastic sheets to allow light into the barn. The roof of the barns is insulated with metal sheeting with large doors of a metal frame with wood and glass windows. Barns are split into three main sections: a central bedded area and two concrete corridors on each side with metal frame wooden feeders along the corridors. Animals always have free access to indoor and outdoor paddocks. Most donkeys are bedded on straw, and their diet is fiber-based, with straw and hay; a vitamin and mineral balancer is added during the winter period when the equids have no access to pasture.

### 2.3. Elements of the IPM Strategy

The IPM strategy, conducted over a minimum of 16 months, involved a combination of permanent trapping of adult *S. calcitrans,* the release of biological parasitoids and predators, the removal of bedstraw, and the protection and treatment of lesions in susceptible donkeys. The aim was to diminish the population of troublesome stable flies, mitigate the nuisance, and reduce skin lesions in animals. The defensive behavior of equids against *S. calcitrans* bites was used to evaluate the impact of the different measures of the IPM strategy. The ultimate objective was to enhance the welfare of animals.

#### 2.3.1. Trapping of *S. calcitrans* Flies

A total of 21 Stomoxycc^®^ traps (Alcochem Hygiene, Zeilmaker 4, 3861 SM Nijkerk, The Netherlands) were installed, deployed, and scattered at different key points across the farm holding (Figure 1B). This trap, available on the market, was recently designed based on the Vavoua model [21]. However, it features a sturdier structure, and the original blue and black fabric was replaced with shiny plastic material. This modification follows the same mechanism, relying on the visual cues provided by the blue and black colors. The trap consisted of a monoconic trap of a cone of mosquito netting attached to three screens joined together at the angles of 120°; the central part of each screen is black, and the outer part is a blue frame (peak of reflectance around 460 nm) (Figure 1C). It can be placed and held into the ground with a stake. The size of the trap is 160 cm (high) and 92 cm (diameter). The plastic anti-UV-catch container placed on top of the trap was filled up with water and soap. Up to 19 traps were deployed in the farm holdings, and two traps were placed on another nearby farm without any control measures, as a control (Figure 1B). The 21 Stomoxycc^®^ traps ran continuously from July 2022 to October 2023, serving as a comprehensive trapping strategy to reduce stable fly adults throughout the year. Additionally, only three of these traps were selected randomly (nº 4, 8, and 13) along with the two controls (nº 20 and 21) to monitor the presence of *Stomoxys* flies. This selection was restricted due to limited manpower. These five traps were visited fortnightly over the mentioned period. During each visit, the contents were emptied (discarded), and then monitoring traps were left operational for 48 h. Subsequently, the collected content was retrieved and analyzed in the laboratory using a stereomicroscope. Therefore, all traps worked by continuously collecting flies (massive trapping) non-stop over the 16-month period. However, every 15 days, a total of five traps were selected to monitor the population dynamics of *Stomoxys* flies. Stable flies (*S. calcitrans*) were identified based on their unique features (a long, stout black proboscis and short maxillary palpi), whereas other insects were identified with different levels of accuracy. The remaining traps were also emptied (and content discarded) on a weekly basis in order not to accumulate too many specimens.

#### 2.3.2. Parasitoids and Predator Agents Release

Three distinct species of biological control agents were used on the farm to manage *S. calcitrans*. These include the predatory mite *M. robustulus* and two parasitoids: *Spalangia cameroni* Perkins and *Muscidifurax raptor* Girault and Sanders (Hymenoptera, Pteromalidae). Biological parasites were released on the farm as part of protocols for controlling immature stages, aiming to establish sustainable reductions in fly populations [5,6,7]. Previous studies have indicated that *S. cameroni* and *M. raptor* successfully reproduced on both fly species, *S. calcitrans* and *Musca domestica*, in dairy farming [22,23,24]. Azevedo et al. [20] have already evaluated the predation capacity of the predatory mites from the Macrochelidae family on fly eggs and neonates.

**Figure 2:**
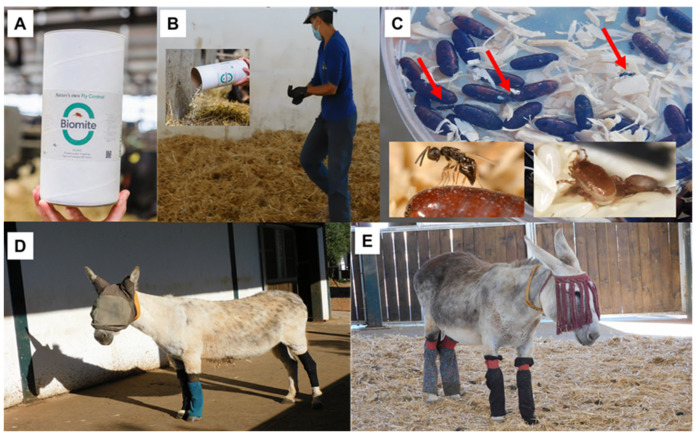
Summary of some management actions conducted in The Donkey Sanctuary (Western Spain). (**A**,**B**) Biological control agents used in this IPM strategy and hand release on the bedding of the barns (**C**). Examples of some specimens of *Spalangia cameroni* emerged from pupae in the laboratory (red arrows) and detailed pictures of *Spalangia cameroni* and predatory acari *Macrocheles robustulus*. (**D**,**E**) Examples of some donkeys using face masks and mesh leggings as protective measures against *Stomoxys* bites.

Parasitic wasps and predatory mites were packaged in small cardboard containers labeled Biowasps^®^ and Biomite^®^, respectively. From 15 June 2022 to 1 September 2023, a total of 14 releases were carried out in the farm (6 in 2022 and 8 in 2023) (Table 1), presumptively involving an estimated 2,200,000 specimens of predatory mites (*M. robustulus*) and 1,316,000 parasitic wasps (*S. cameroni* and *M. raptor,* with 50% of each species) during the stable fly season. The biological control agents were manually spread out over the seven donkey beds in a uniform manner by the sanctuary staff (Figure 2A,B). These predators live in the litter areas and manure, where they naturally sustain their numbers in the presence of their hosts.

Biomite^®^ and Biowasp^®^ are safe to use and have no negative impact on animals and farmers. The release consists of spotting the wet part of the bedding, where generally immature flies accumulate to spread manually the beneficials. Releases occur every 2 to 4 weeks, depending on fly density pressure and/or logistical circumstances (Table 1). Due to reasons beyond our control, we were not authorized to leave “free zones” in the farm environments, which prevented us from performing comparison tests, as the welfare of the donkeys prevailed, and the entire barn beds were treated with parasitoids. The success in the performance of the control agents was verified in three moments (12 June 2023, 27 July 2023, and 5 September 2023) by collecting fly pupae in the bedding (Figure 2C). Sampling revealed a limited fly hatching rate, hindered by parasitoid effect, including reproduction and host feeding.

#### 2.3.3. Straw Bed Management

All the barns were bedded on straw except barn number 2, which contained wood shavings. Approximately 136 m^3^ of bedding from the barns was mechanically removed at least every 15 days from March to November using a tractor. For the rest of the year, bedding was removed at least once a month. The content was then driven to the dung heap and emptied in a pile near fly traps 16–19. The dung heap was removed from the farm twice a year. Barns were disinfected when removing bedding using an antibacterial, antiviral, and antifungal product containing Didecyl dimethyl ammonium chloride 6.9% (SANITAS^®^) or another product containing primarily biphenyl-2-ol 4.0% and chlorocresol 0.90% (Zotal^®^). Feeders were also disinfected at least twice a month.

#### 2.3.4. Monitoring Defensive Behaviors of Equids

To investigate the fly avoidance behavior of the animals, video recordings of equids were captured by using the camera of a standard Android device. As equids were roaming freely in the farm holdings, our approach was adopted to prevent any interference with their natural behavior. Thus, the recording distance varied from a few meters to several meters, given the inherent challenge of controlling this variable. Video recordings (ca. 3 min duration) were always carried out from 11.00 to 13.00 from August 2022 to October 2023. The recordings featured equids located in specific areas of the farm environment, resulting in a total of 235 recordings over 15 months. These locations remained constant, situated near the traps; however, the selection of the animals was randomized to mitigate potential effects related to age, race, and individual sensitivity [11,13]. Between 15 and 18 animals were selected once a month and observed from one side. Subsequently, five different defensive behaviors (Table 2) were recorded by one of the authors based on stable fly-defensive behaviors described in previous literature [11] (and reference therein). Defensive movements such as head-neck shake and front and back leg stamps were scored, and a numerical value (scale 0–4) was assigned (calculated as the number of movements divided by the total recording time of the video). In the case of ear and tail swishes, these were calculated according to the frequency of movement using the following scale: 4 (very often), 3 (often), 2 (now and then), 1 (low), and 0 (not observed). Then, a quantitative index was calculated for each animal based on the sum of the scores of the five defensive movements. Pooling these defensive behaviors ensures that the combined data represent a more comprehensive representation of overall fly-repelling behavior [11]. Fly-repelling behaviors provide direct information on stable fly attack rates [11]. Panniculus reflex (skin twitches) was not recorded due to cases where the distance did not allow observation of such descriptors. Additionally, small movements, likely in response to other insect activity, were excluded. Equines often executed small headshakes or throws that were not recorded as valid [11].

#### 2.3.5. Assessment of Welfare and Protection Measures in Donkeys

A veterinary team assessed the welfare status of those animals showing major severity in their skin lesions (in total 14 donkeys) caused by *S. calcitrans* bites from May 2021 until November 2023. Targeted animals were inspected monthly to assess the affected area (face, chest, and number of extremities) and the severity of the skin lesions (0: without lesions, 1: very slight lesions, 2: well-defined lesions, 3: moderate lesions, 4: intense lesions, 5: severe lesions). In addition, an index, called extension, was calculated with the total number of anatomical parts affected in each animal. The values ranged from 0 to 4, thus allowing us to show the relationship with the severity in the same ranges.

Moreover, the combination of multiple protective measures to prevent direct contact and bites, such as protective rugs, fly boots, face masks, and mesh leggings (Figure 2D,E), were used in those 14 donkeys and others if necessary (this did not affect the interpretation of the results), as well as topical repellents, that can significantly minimize the risk of *Stomoxys* fly bites and ensure the well-being of equines [6,7]. As flies tend to target the lower parts of a donkey’s legs, leggings made of mesh, cotton, or other fly-repelling materials can be used as a protective measure. Thus, pro-wrap bandages with cotton wool underneath were used as a barrier to protect legs; however, they were found to be non-breathable and were changed to cotton wool leggings. Fly masks and anti-fly fringes were used for the protection of the face and lacrimal area, allowing the donkey to see and breathe comfortably while protecting sensitive areas. In some cases, lightweight fly sheets made of mesh or similar materials were used to cover the donkey’s body from neck to tail and fly rugs to protect the pectoral area. Please note that, unfortunately, the authors were not able to make decisions on how to implement the measures of the animals as they were in charge of the vet team. At all times, the welfare of the donkeys prevailed over the study guidelines.

Regarding chemical protection, deltamethrin pour-on (BUTOX, emulsion pour-on^®^) was applied twice a year, in early spring and during summer. On peaks of abundance, for the most affected donkeys, anti-feeding and repellent products (Tri-Tec 14™ and Pody^®^ care) were sprayed on donkey bodies to repel or kill *S. calcitrans* flies by contact. These products contained ingredients like pyrethrin, permethrin, and tetramethrin.

#### 2.3.6. Statistical Analyses

The number of *S. calcitrans* captured by trapping was compared by date and trap with full-factorial general linear models (GLM). The same model was conducted to detect differences in the frequency of non-target fauna captured by trapping, including the number of different groups of non-target fauna as a weighted factor. When significant differences between dates and traps were detected, Duncan’s post hoc test was carried out. Non-target fauna was classified into groups according to abundance, ecological interest, and sanitary interest (Order: Diptera, Hymenoptera, Lepidoptera, and others; infraorder level or at family level).

We analyzed climatic variables over a period of three years (2021–2023) to detect possible differences that could influence the development of *Stomoxys* populations in our study area and to relate these possible climatic changes to the differences in fly populations observed in the trapping. Normality was assessed with the Shapiro–Wilk test and analyzed for significant differences by year and date with the Kruskal–Wallis (KW) test, using the Mann–Whitney U test as a post hoc to compare between two categories. Differences among the average temperature, precipitation, and sunshine hours by month were analyzed using the KW test.

To evaluate the defensive behaviors, contingency tables were made with the date and the score for each of the defensive movements and then were analyzed by Pearson’s Chi-square test (when less than 20% of expected values were lower than 5, in other cases, the likelihood ratio test was applied). Differences between the date and the qualitative, quantitative index were assessed by the KW test plus Mann–Whitney’s U post hoc test to evaluate the differences between dates. Contingency tables were made corresponding to the cross between the scores of each of the defensive movements and were analyzed using Somers’ d correlation test. To assess the relationship between the number of *S. calcitrans* captured by trapping and the defensive behaviors of the equids, Spearman’s correlation test was performed.

Contingency tables were also used to evaluate the data with respect to the anatomical part affected by the lesion, the extent of the lesion and its severity (prevalence in each moment was calculated using this data and assuming a population at risk of 141 animals). Confidence intervals of frequencies were calculated using Wilson’s Score method. To assess whether there was a correlation between the extension and the severity of the lesions, correlation tables were made using the Somers’ d test.

Statistical analyses were performed using SPSS 19.0 for Windows (IBM, Armonk, NY, USA). Figures were generated with Microsoft Excel 2013.

## 3. Results

The comprehensive study conducted at The Donkey Sanctuary encompassed various aspects related to stable fly population dynamics and collection of non-target fauna, impact of climatic variables, animal manure removal, release of biological control agents, assessment of defensive behaviors of equines, as well as body protection and health of animals. Notably, five Stomoxycc^®^ traps strategically placed collected substantial numbers of stable fly specimens in 52 days of captures from July 2022 to October 2023, revealing consistent activity of *S. calcitrans* with peaks of abundance in mid-summer and autumn. These traps were also shown to be efficient in collecting other groups with medical and veterinary importance. Climatic analyses exhibited significant differences in temperature and sunshine hours over the years. Our trapping system inadvertently collected non-target beneficial insects but in low numbers. Equids exhibited five mainly avoidance behaviors that were higher in 2022 than in 2023, with fly bother peaking in September 2022. Additionally, the health assessment revealed a higher incidence of lesions on equids’ legs during summer months, progressing to various severity grades, with a strong correlation between extension and severity. The study provides valuable insights into the interplay of environmental factors, insect dynamics, and equid health, suggesting potential avenues for integrated pest management and protective measures.

### 3.1. Monitoring Population Dynamics of Stable Flies

**Figure 3:**
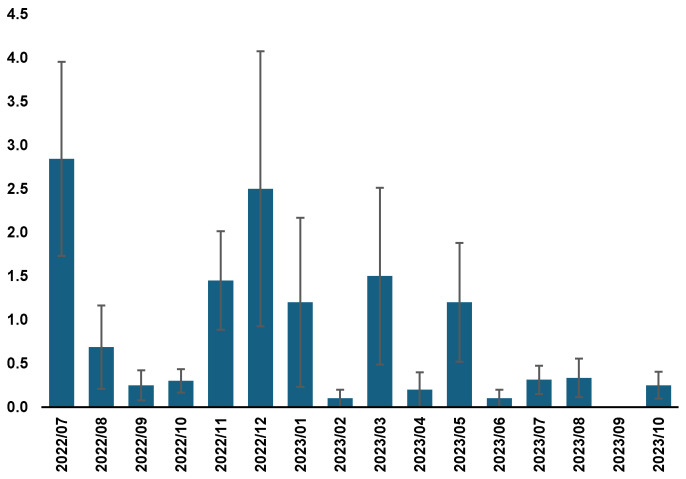
Population dynamics of *Stomoxys calcitrans* caught per month in five Stomoxycc^®^ traps in The Donkey Sanctuary (Western Spain) during 2022–2023.

The five Stomoxycc^®^ traps strategically positioned across The Donkey Sanctuary collected a total of 631 specimens of adult stable flies. Stable fly collections were consistently active throughout the entire sampling period, displaying a variable pattern, with a remarkable peak of activity in mid-summer (July–August) and a secondary peak in autumn (November) (Figure 3). In July and August 2022, 2.8 and 0.68 stable flies/trap/day were captured, compared to 0.31 and 0.33, respectively, in 2023. Additionally, low but consistent numbers of stable flies were also collected during the winter season (Figure 3). The GLM analysis (*p* < 0.01) showed significant differences by date in the number of *S. calcitrans* trapped over time, with the months of July and December 2022 showing significant differences with respect to the rest of the months (Duncan’s post hoc test, *p* ≤ 0.05) (Supplementary Table S1). The GLM also showed significant differences in the abundance of flies by traps (*p* ≤ 0.001), with trap nº 4 being the one that collected the highest number of *S. calcitrans* (Duncan’s post hoc test, *p* ≤ 0.05) (Supplementary Table S2). Control traps recorded very low numbers of stable flies (*n* = 14), which indirectly hampered their use as effective control to evaluate our IPM strategy.

Our study displayed 19 collection traps (excluding controls) working continuously over 16 months as a reduction approach. Considering the mean catches/trap/day obtained in three monitoring traps, we estimate that between 3561 and 15,860 (depending on the trap performance) *S. calcitrans* flies were collected and killed by our trapping system over the period of the IPM.

### 3.2. Analysis of Climatic Variables

Significant differences were observed in the variable temperature (T) over time (2021–2023). Minimum T (*p* = 0.001) in 2021 (10.57 °C), 2022 (11.40 °C), and 2023 (12.17 °C), and maximum T (*p* < 0.001) in 2021 (23.64 °C), 2022 (24.76 °C) and 2023 (26.39 °C). There were no significant differences (*p* = 0.219) in precipitation (1.61 L/m^2^ in 2021, 1.07 in 2022, and 0.73 in 2023), but a trend towards less rainfall over time was observed. Significant differences (*p* = 0.008) were found in sunshine hours (8.52 h/day in 2021, 8.55 in 2022, and 9.50 in 2023) (Supplementary Figures S1–S4).

### 3.3. Non-Target Fauna

Collections made in five traps biweekly over a 16-month period (48 h collections) accounted for a total of 1423 insects. The most abundant order was Diptera (*n* = 1143, 81.2%), followed by Hymenoptera (*n* = 136, 9.6%), Lepidoptera (*n* = 101, 7.6%), and seven other Orders computed to a lesser extent. Notably, the Brachycera suborder (including families Muscidae, Anthomyiidae, Sarcophagidae, Tachinidae, and Calliphoridae) dominated (*n* = 737), with substantial collections of *M. domestica* and *Musca autumnalis*. Within non-target fauna with significance as pollinators or beneficial insects, the family Syrphidae (genera *Eupeodes, Eumerus, Syritta,* and *Sphaerophoria*) was the most affected (*n* = 155), followed by Apidae, Colletidae, and Andrenidae (*n* = 47) and other families in small numbers (Bombyliidae, Conopidae, Vespidae, and Hesperidae).

The abundance of insect collections varied depending on the season and trap. September–October (2022) comprised the highest number of specimens (*n* = 264), followed by April–May (2023) (*n* = 202) of non-target fauna, while the summer months (July–August 2023) recorded the lowest numbers (*n* = 49) along with winter months (December 2022–February 2023), (*n* = 87). By date, Culicidae (*p* = 0.012), other Diptera (*p* < 0.001), other Hymenoptera (*p* = 0.003), and Syrphidae (*p* < 0.001) showed significant differences in the GLMs. By trap, significant differences were found for Muscomorpha (*p* = 0.010) and Culicidae (*p* = 0.030) in the GLMs (Supplementary Tables S3 and S4).

In addition to stable flies, various other interesting insect groups of medical and veterinary interest were also collected: Mosquitoes (Diptera: Culicidae) belonging to the genera *Culiseta* and *Culex (n* = 33), horse flies, and clegs (Diptera: Tabanidae) such as *Tabanus glaucopis* (*n* = 3), *Tabanus bovinus* (*n* = 1) and *Haematopota* sp. (*n* = 7), horn flies *Haematobia irritans* (Diptera: Muscidae) (*n* = 7), sand flies (Diptera: Psychodidae) (*n* = 5) and the horse bot fly *Gasterophilus intestinalis* (Diptera: Oestridae) (*n* = 2).

### 3.4. Defensive Behaviors of Equids

**Figure 4:**
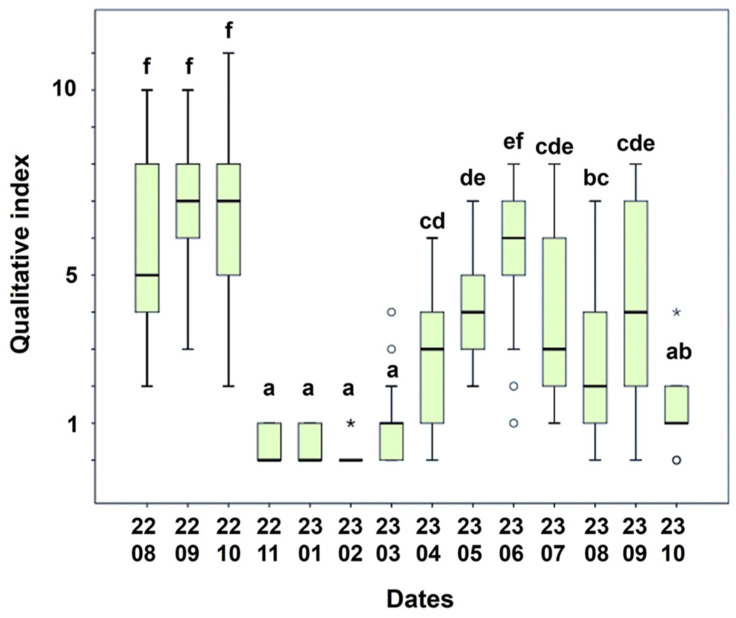
Box-plot graph showing the qualitative index (sum of the five defensive behaviors) from video recordings on equids over time conducted in The Donkey Sanctuary (Western Spain). Different superscripts for each date indicate significant differences according to Duncan’s post hoc test (*p* ≤ 0.05). The circles indicate extreme values and the * indicate outlier values.

**Figure 5:**
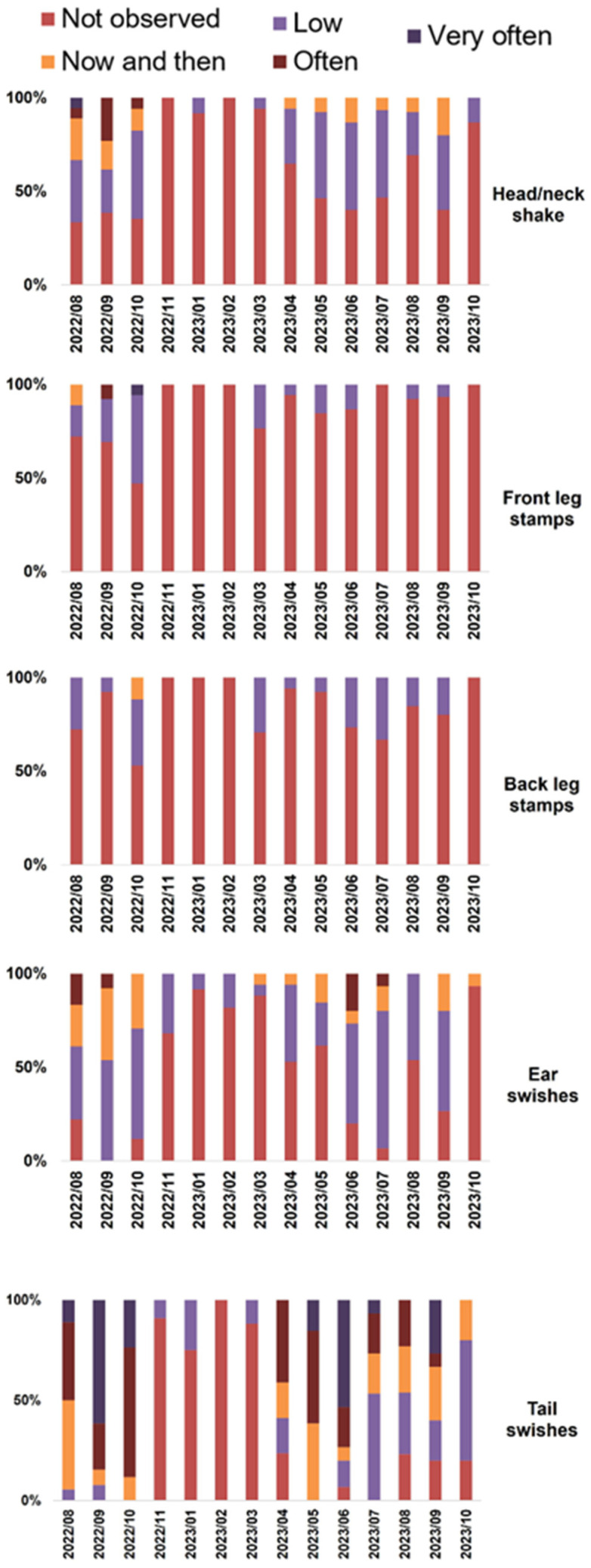
Summary of the five fly-repelling reactions (head/neck shake, front leg stamps, back leg stamp, ear, and tail swishes) recorded from equids over time in The Donkey Sanctuary (Western Spain).

This study presents a detailed analysis of fly-deterrent and fly-induced reactions in equids. Head tossing and foot stamping were less frequent than ear and tail flicks. The Spearman’s correlation test between catches of *S. calcitrans* and fly-repelling behaviors showed to be not statistically different (*p* = 0.399). The decrease in the qualitative indices of the annoyances manifested by the equids from the summer of 2022 in relation to those reported in the summer of 2023 (Figure 4 and Figure 5) is consistent with the parallel decrease in captures in the traps. The qualitative index showed significant differences with respect to the month (*p* < 0.001). The months of August, September, and October 2022 were the months with the highest index, followed by the summer months of 2023 (Figure 4). This data indicates that September 2022 consistently denotes the peak of fly bother. Based on previous scores and index, we inferred a decrease in the biting nuisance seasonal activity in November 2022 and October 2023, which afterward remained at a low level during the winter months (December, January, February, and March), and an increase started in April 2023 (Figure 4 and Figure 5).

Significant differences in four defensive movement scores compared to the year-earlier period were found: Ear swishes (*p* < 0.01), head/neck shake (*p* < 0.01), front leg stamp (*p* = 0.228), back leg stamp (*p* = 0.007) and tail swishes (*p* < 0.01) (Figure 5) and all of them remained high in summer–autumn months. Tail swishes were the defensive action with the highest frequency.

The body’s defensive movements are significantly correlated but with low values, so they must be examined. The most correlated movements are head–ear, ear–tail, and head–tail (Supplementary Table S5).

### 3.5. Health of Animals and Protective Measures

**Figure 6:**
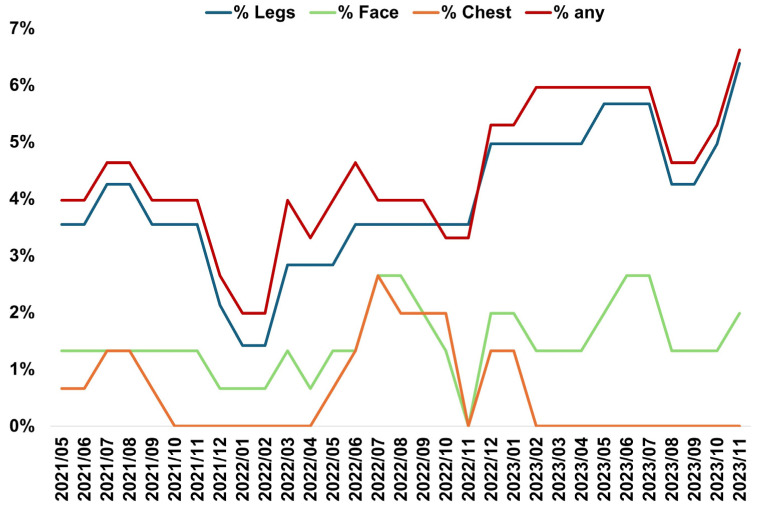
Indicator of lesions in donkeys (*n* = 14) caused by *S. calcitrans* bites according to the an-atomical part over time in the Donkey Sanctuary (Western Spain). Percentage is calculated as the number of animals affected divided into the total for the period May 2021 to November 2023.

Fourteen animals (out of 141) presented visible clinical lesions during the study period. The prevalence (nº of animals affected/total) of lesions by anatomical area throughout the study period is shown in Figure 6.

**Figure 7:**
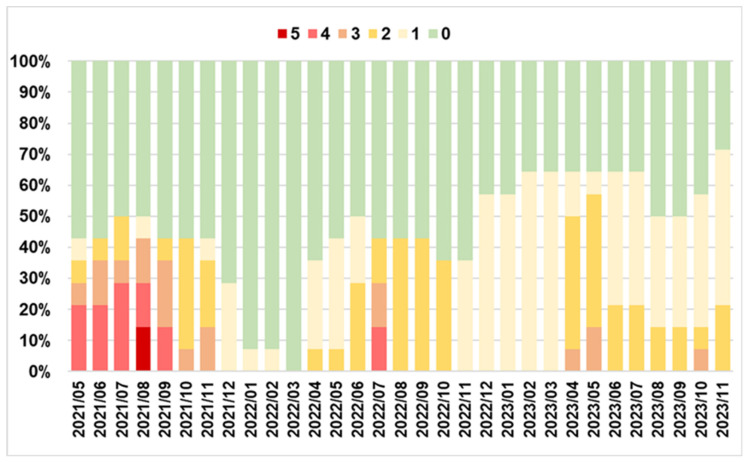
Degree of severity of *S. calcitrans* bite injuries in donkeys (*n* = 14) over time (May 2021 to November 2023) in The Donkey Sanctuary (Western Spain). Scale: 0: without lesions, 1: very slight lesions, 2: well-defined lesions, 3: moderate lesions, 4: intense lesions, 5: severe lesions.

The legs were the anatomical part with the highest incidence of lesions. Summer months (July–September) showed the highest incidence of lesions, and winter months the lowest (January and February) (Figure 6, Table S6). The chest (*p* = 0.030) was the only anatomical area with significant differences by date. Significant differences were observed between date and degree of severity (*p* < 0.001), being the months between May and September 2021 together with September 2022, the dates that have reached the maximum grade 5. The date with the most severe lesions (grade 5) was August 2021 (Figure 7). Afterward, there is a clear declining trend in the degree of severity with respect to time, with severity increasing from grade 2 to 3 always during the month of April and remaining at those levels until October-November each year (Figure 7).

There is a significant correlation between the extension and severity of lesions, and the correlation is high (Somers’ d = 0.820; *p* < 0.001) (Supplementary Figure S5).

## 4. Discussion

The present article proposed a reasonable IPM approach to manage stable flies, incorporating the synergy of cultural, mechanical–physical measures and biological control agents. Managing *S. calcitrans* flies is challenging due to their dispersal behavior, and the fact that they are not resident on the hosts. Individually, none of the control measures tested in the literature has yielded satisfactory results; therefore, integration of multiple actions is needed [6,7]. Our study represents the first attempt, at the European level, to integrate several ecological interventions aimed at improving the welfare of 141 equines with severe nuisance inflicted by stable fly bites. The IPM strategy implemented in The Donkey Sanctuary in western Spain provides interesting and new data on stable fly dynamics and defensive behaviors of the equines, contributing to providing guidelines to achieve potential improvements in the management and control of this biting fly nuisance in the future. To our knowledge, this study constitutes the unique work published on donkey behavioral responses to fly attacks and assessment of body injuries in Europe, as previous attempts were made in cattle from America [11,12,13]. It is also the first time a combination of both predatory mite and parasitic wasp control agents has been tested. The abundance of stable flies declined from 2022 (the start of IPM) to 2023 and the welfare of the equines was also moderately improved over this period. However, we cannot attribute the drop in the number of fly catches exclusively to the implementation of the IPM strategy because many factors could have also contributed to this reduction. For example, we found that the climatic conditions (temperature, precipitation, and sunshine hour) varied among the studied years, but the recorded variations do not seem to indicate unfavorable conditions for the flight, behavior, or reproduction of this species in 2023. In addition, our control traps, unfortunately, did not display substantial numbers for meaningful comparison, thus complicating the interpretation. The heightened activity of flies in the vicinity of active animal facilities and the influence of temperature and precipitation, which might explain most of the seasonal trap catch variance [25,26,27], represent key factors that should be considered.

The dynamic population of stable flies has been extensively studied worldwide, including research from Europe [28,29,30]. In equine centers of the UK, the abundance of *S. calcitrans* has been shown to have a strong seasonal pattern, with the population increasing gradually over the summer months and peaking in late August/September (E. Barrio, personal communication), which is in line with our results. In Slovakia, a large peak in the seasonal activity of stable flies at the end of summer and a second, smaller peak just before the end of the flight season was recorded [28]. In dairy farms in Denmark, the peak of abundance occurred in July [29]. In France, a bimodal pattern of stable fly population dynamics was observed, with both peaks occurring in early summer and fall [30]. These authors indicated the non-existent activity of *Stomoxys* outdoors in winter, which contrasts with other studies, where small numbers of adult stable flies can be observed outdoors on warm winter days, aligning with our results [11,31]. Flight abundance, as shown in [28,29,30], varied between sites, and multiple factors, such as temperature, population size, light conditions, air humidity, precipitations, habitat, or animal management practices, may strongly influence local abundance. It is feasible to attribute the decrease at the end of the summer, particularly in the year 2022, to warm and dry weather, with a second small population peak in late fall because of the return of precipitation and warm weather.

Several studies have suggested that massive trapping might be an effective management strategy [6,7]. Regarding the effectiveness of traps, the Stomoxycc^®^ model has been formally tested and compared with other models recently in France [32] and in Thailand (unpublished). Stomoxycc^®^ traps captured more flies than the other two models (Vavoua trap and horizontal trap) and less non-target fauna [32]. In our study, traps were also shown to be efficient in collecting adult stable flies. We would like to note some future recommendations regarding the use of these traps under field conditions. The attraction mechanism of these traps relies on the reflectance of light on the black and blue material [19,33,34]. Small changes in color have a strong impact on the effectiveness of traps. For this reason, over time, these traps tend to be covered with dust and dirty, particularly in places with a flow of vehicles on unpaved roads. It seems reasonable that the effectiveness of these traps might be hampered, and weekly cleaning is mandatory to guarantee their optimal effectiveness. Another aspect to be improved in the future is the long viability of the traps and they require some modifications to improve the quality. The jar collector made of plastic suffered cracks and breaks in most of the traps after 12–14 months of use. This problem has also been observed in similar traps (model H-traps designed for tabanids), which might reflect the extreme temperature typical in Mediterranean countries in the summer season.

Traps should be efficient both in collecting targeted species and be innocuous or have little impact on non-target fauna to be environmentally ground. The collection of insect pollinators, sensitive species, and beneficial fauna in traps is not acceptable from an environmental perspective, and more sustainable alternatives are needed. Our study showed differences in the abundance of some non-target fauna based on trap position and date. Despite non-target fauna not being very high in our study, traps should be shifted to other habitats where non-target fauna might be present in low numbers and avoid some dates that may severely impact beneficial groups. Previous studies have shown that blue screens collected few non-target fauna [34]. However, blue sticky traps have shown severe negative effects on butterflies in France and Costa Rica (Duvallet, unpublished). Research to improve trap designs and selectivity minimizing the impact on native entomofauna is essential [35,36,37]. We are exploring ways to make the trap system more selective, possibly by incorporating a filter at the opening of the collection bin and adding small holes in the lid for smaller species to escape. There is an urgent need to improve trap designs by leveraging advancements in trapping methods to enhance selectivity. The advantage of this trap is that, apart from trapping stable flies, it also demonstrates effectiveness in collecting a wide range of insects with medical and veterinary significance. This is valuable not only for massive trapping but also for monitoring purposes.

Biological control methods have gained popularity in pest management within the last few decades. Over 150 natural enemy species are available for this purpose. This approach has proven effective in agricultural settings, reducing the need for chemical insecticides [37]. The use of natural enemies like parasitic wasps to control pests like *Stomoxys* flies and the release of parasitic wasps and predatory acari into livestock facilities can significantly reduce the number of stable flies, thereby improving animal welfare [6,7]. We corroborated three times during the studied time that the parasitic wasps emerged from pupae of *S. calcitrans* in manure-soiled straw bedding, thus probing their viability under the given scenario. Previous studies have extensively demonstrated the effectiveness of parasitic wasps in reducing *Stomoxys* populations by 10–30% [6,29], indicating their potential as a viable approach for enhancing welfare.

As stable flies lay their eggs in decaying organic matter, such as manure, bedding materials often provide suitable breeding sites. Thus, regular removal of soiled bedding disrupts the stable fly life cycle by eliminating the substrate where female flies deposit their eggs. Stable-fly development was reduced by >90% when crop residues were treated, but the authors highlighted the importance of conducting the spraying either prior to or immediately after the incorporation of the residue into the soil [38]. Sanitation or on-farm hygiene are the simplest methods to employ, which include regular removal of animal manures and soiled animal bedding [6]. However, nobody has assessed the quantitative impact of this approach. Our IPM strategy also contributed to this strategy by removing the animal bedding from barns on a regular basis, thus contributing to eliminating the presence of immature stages of flies in the animal barns, and subsequently from the farm.

Insect-repelling movements of the tail, ears, head, and feet are widespread in mammals and effective in reducing bites [39,40]. The frequency of these fly-deterrent behaviors might serve as an indicator of the stable fly attack rate [11,41]. It is important to determine which defensive movement provides a better measure of fly avoidance behaviors. However, fly-repelling behavior might not solely respond to *S. calcitrans* bites, as some defensive movements, such as tail flicks, might indicate irritation or the presence of “fly nuisance” [42]. The deterrent response also might not respond to the fly abundance [12]. By pooling fly-induced reactions, we ensured that the pooled data provided a better picture of the genuine annoyance, as one of the specific movements alone might not serve as an indicator. Our study did not find a correlation between *Stomoxys* abundance and fly-repelling behavior in equids, which is reported in other studies. In cattle, a correlation was found between alighted stable flies and movements [12], but some kind of habituation was found in other studies. Ratios of leg stamps and head throws to fly numbers might cause habituation to pain associated with fly biting [11]. At the same time, Dougherty et al. [43] observed declining rates of behaviors over 1 h periods that might involve habituation. This trend has also been observed in equids in a Donkey Sanctuary from the UK after a 12-week period subjected to *Stomoxys* attack, and habituation was recorded. However, it is interesting to note that previous studies on cattle have shown that tail flicks, though not effective for repelling *Stomoxys,* were the easiest to quantify and monitor pest intensity [10]. The fact that tail swishing was the most frequently occurring behavior was also observed in horses [9]. However, these energetically low-cost behaviors were observed to occur in response to both biting insects and non-biting insects, whereas the more energy-demanding behaviors (such as head and leg movement) occurred mainly in response to biting flies [9].

Detailed information on equine lesions by *Stomoxys* bites is limited. The preferred biting sites of *S. calcitrans* depend on the host [44]. In donkeys, the lesions infringed by stable flies are concentrated primarily on the extremities and, to a lesser extent, on the face and/or chest [45]. Severity and extent are highly correlated so that one could dispense with calculating e.g., extent. Our study showed that the number of animals affected did not change over time, but the severity of the lesions declined after the implementation of the IPM strategy. This improvement might be presumably attributed to the decline in the number of stable flies but particularly to the health care (topical treatments) and protective measures. The benefits of physical barriers (mesh legging and/or leg bands) against stable flies to reduced foot stomp behavior in both horses and dairy cows have been reported in other studies [14,46].

Most previous studies have described the principles of the IPM approaches and/or push-pull strategies [6,7,47]; however, very few studies have presented case studies or tested these methodologies under field conditions [1,48]. To the best of our knowledge, we provide one of the most comprehensive examples of non-toxic IPM programs published so far.

## 5. Conclusions

An all-encompassing and environmentally friendly approach has been proposed to tackle the challenge of controlling stable fly populations. Managing these flies has proven to be one of the most challenging tasks in pest control. This study is a pioneering effort in Europe, offering the most extensive trial attempt control management conducted to date on stable flies. Significant progress was made in enhancing sanitary conditions among equids. However, the continuous persistent outdoor activity of stable flies in this region throughout the year poses a considerable hurdle in curbing this pest. Employing a combination of these methods and ensuring consistent efforts in pest control can prove instrumental in managing stable fly populations. Integrated pest management strategies, which amalgamate various control measures, consistently demonstrate the most successful outcomes in combatting resilient pests like the *S. calcitrans* flies.

## Figures and Tables

**Table 1:** Summary of the chronological release (by week) of beneficial arthropods in 2022–2023 in The Donkey Sanctuary (Western Spain).

Biological Control Species	Weeks of Release								
		22	24	26	28	30	32	34	36
*Macrocheles robustulus*	2022			X		X		X	
	2023	X	X	X	X	X	X	X	X
*Spalangia cameroni*	2022			X	X	X	X	X	X
	2023	X	X	X	X	X	X	X	X
*Muscidifurax raptor*	2022			X	X	X	X	X	X
	2023	X	X	X	X	X	X	X	X

**Table 2:** Ethogram of fly-repelling behaviors recorded in equines of The Donkey Sanctuary (Western Spain) from August 2022 to November 2023.

Fly Repelling Behaviors	Description	
Tail swish	Flicks of the tail: movement of the tail from its straight-down resting position to the side	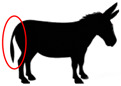
Head throw, neck shake, and/or nose rub	Shaking or tossing of the head or neck and/or rubbing of the nose against the leg or other body part (usually the bitten area of the body)	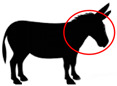
Front leg stamp	Lifting of a hoof and stamping it back down	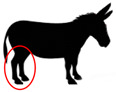
Back leg stamp	Lifting of a hoof and stamping it back down	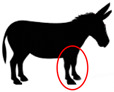
Ear swish	Swish of the ears	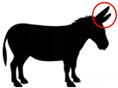

## Data Availability

The original contributions presented in the study are included in the article/supplementary material, further inquiries can be directed to the corresponding author.

## Supplementary Materials

The following supporting information can be downloaded at: https://www.mdpi.com/article/10.3390/insects15040222/s1, Figure S1A. Maximum annual temperatures recorded from the meteorological station in Jerez de Los Caballeros, located at 19 km longitudinal distance from The Donkey Sanctuary (Western Spain) for the period 2021–2023. Figure S1B. Maximum monthly temperatures recorded from the meteorological station in Jerez de Los Caballeros, located at 19 km longitudinal distance from The Donkey Sanctuary (Western Spain) for the period 2021–2023. Figure S2A. Minimum annual temperatures recorded from the meteorological station in Jerez de Los Caballeros, located at 19 km longitudinal distance from The Donkey Sanctuary (Western Spain) for the period 2021–2023. Figure S2B. Minimum monthly temperatures recorded from the meteorological station in Jerez de Los Caballeros, located at 19 km longitudinal distance from The Donkey Sanctuary (Western Spain) for the period 2021–2023. Figure S3A. Annual recorded precipitation from the meteorological station in Jerez de Los Caballeros, located at 19 km longitudinal distance from The Donkey Sanctuary (Western Spain) for the period 2021–2023. Figure S3B. Monthly recorded precipitation from the meteorological station in Jerez de Los Caballeros, located at 19 km longitudinal distance from The Donkey Sanctuary (Western Spain) for the period 2021–2023. Figure S4A. Daylight hours recorded annually from the meteorological station in Jerez de Los Caballeros, located at 19 km longitudinal distance from The Donkey Sanctuary (Western Spain) for the period 2021–2023. Figure S4B. Daylight hours recorded monthly from the meteorological station in Jerez de Los Caballeros, located at 19 km longitudinal distance from The Donkey Sanctuary (Western Spain) for the period 2021–2023. Figure S5. Correlation between extension and severity of skin lesions in donkeys caused by *Stomoxys calcitrans* bites in The Donkey Sanctuary (Western Spain). Table S1. Mean ± standard deviation of the catches of *Stomoxys calcitrans* in Stomoxycc^®^ traps by date in The Donkey Sanctuary (Western Spain). Table S2. Mean ± standard deviation of the catches of *Stomoxys calcitrans* in Stomoxycc^®^ traps by trap in The Donkey Sanctuary (Western Spain). Table S3. Mean ± standard deviation of the catches of non-target fauna in Stomoxycc^®^ traps by date in The Donkey Sanctuary (Western Spain). Table S4. Mean ± standard deviation of the catches of non-target fauna in Stomoxycc^®^ traps by trap in The Donkey Sanctuary (Western Spain). Table S5. Somer’s d correlation test between the five defensive movements of equids against bites of *Stomoxys calcitrans* in The Donkey Sanctuary (Western Spain). Table S6. Indicator of lesions in donkeys (*n* = 141) caused by *Stomoxys calcitrans* bites according to the anatomical part over time in the Donkey Sanctuary (Western Spain). Percentage is calculated as the number of animals affected divided into the total, and 95% confidence interval is calculated using the Wilson’s Score method. For the period May 2021 to November 2023.
