# Supplementary material for: An Integrated Pest Management Strategy Approach for the Management of the Stable Fly Stomoxys calcitrans (Diptera: Muscidae)
Source: Insects. 2024 Mar 25;15(4):222. doi: 10.3390/insects15040222 (PMC11050470; doi:10.3390/insects15040222)
Supplement: Supplementary file 1 [file insects-15-00222-s001.zip › insects-2848600-supplementary.pdf]

Supplementary material

Table S1. Mean ± standard deviation of the catches of *Stomoxys calcitrans* in Stomoxycc® traps by date in The Donkey Sanctuary (Western Spain).

Year	Month	Catches
2022	7	$2.84^{b} \pm 3.51$
	8	$0.69^{a} \pm 1.51$
	9	$0.25^{a} \pm 0.54$
	10	$0.30^{a} \pm 0.42$
	11	$1.45^{ab} \pm 1.78$
	12	$2.50^{b} \pm 3.51$
2023	1	$1.20^{ab} \pm 2.16$
	2	$0.10^{a} \pm 0.22$
	3	$1.50^{ab} \pm 2.26$
	4	$0.20^{a} \pm 0.44$
	5	$1.20^{ab} \pm 2.15$
	6	$0.10^{a} \pm 0.22$
	7	$0.23^{a} \pm 0.45$
	8	$0.29^{a} \pm 0.66$
	9	$0.00^{a} \pm 0.00$
	10	$0.20^{a} \pm 0.48$
**Total**		**0.81 ± 1.76**

Significance according GLM test, $p = 0.001$. Different superindexes for each date indicate significant differences according to Duncan's post hoc test ($p < 0.05$).

Table S2. Mean ± standard deviation of the catches of *Stomoxys calcitrans* in Stomoxycc® traps by trap in The Donkey Sanctuary (Western Spain).

Trap	Catches
4	$2.14^{c} \pm 2.99$
8	$1.09^{b} \pm 1.73$
13	$0.55^{ab} \pm 0.79$
20	$0.17^{a} \pm 0.42$
21	$0.07^{a} \pm 0.23$
**Total**	**0.81 ±1.76**

Significance according GLM test, $p < 0.001$. Different superindexes for each date indicate significant differences according to Duncan's post hoc test ($p < 0.05$).

Table S3. Mean ± standard deviation of the catches of non-target fauna in Stomoxycc® traps by date in The Donkey Sanctuary (Western Spain).

Year	Month	n	Brachycera	Bombyliidae	Apinae	Culicidae	Lepidoptera	Other Diptera	Other Hymenoptera	Syrphidae
2022	9	10	4.00 ± 3.07	0.00 ± 0.00	0.95 ± 1.77	0.05 ^{a} ± 0.16	0.75 ± 1.03	0.25 ^{a} ± 0.26	0.30 ^{ab} ± 0.54	1.60 ^{cd} ± 1.80
	10	10	6.95 ±12.81	0.00 ± 0.00	0.25 ± 0.42	0.25 ^{ab} ± 0.42	1.70 ± 4.33	0.20 ^{a} ± 0.35	0.00 ^{a} ± 0.00	1.10 ^{abcd} ± 1.35
	11	10	4.05 ± 5.71	0.00 ± 0.00	0.70 ± 1.27	0.10 ^{a} ± 0.21	0.10 ± 0.21	0.20 ^{a} ± 0.42	0.05 ^{a} ± 0.16	0.35 ^{ab} ± 0.47
	12	5	4.70 ± 3.93	0.00 ± 0.00	0.00 ± 0.00	0.00 ^{a}± 0.00	0.20 ± 0.27	0.40 ^{ab} ± 0.42	0.10 ^{a} ± 0.22	0.00 ^{a} ± 0.00
2023	1	5	5.60 ± 4.35	0.00 ± 0.00	0.00 ± 0.00	0.00 ^{a} ± 0.00	0.00 ± 0.00	0.50 ^{ab} ± 0.87	0.10 ^{a} ± 0.22	0.00 ^{a} ± 0.00
	2	5	0.00 ± 0.00	0.00 ± 0.00	0.40 ± 0.42	0.00 ^{a} ± 0.00	0.00 ± 0.00	1.80 ^{cd} ± 0.67	0.00 ^{a} ± 0.00	0.00 ^{a} ± 0.00
	3	5	0.10 ± 0.22	0.00 ± 0.00	0.60 ± 0.89	0.00 ^{a} ± 0.00	0.20 ± 0.27	1.70 ^{cd} ± 0.84	0.60 ^{abc} ± 0.65	0.40 ^{a} ± 0.42
	4	5	2.60 ± 2.48	0.10 ± 0.22	0.40 ± 0.42	0.00 ^{a} ± 0.00	0.40 ± 0.42	2.20 ^{d} ± 1.92	1.00 ^{c} ± 0.50	2.10 ^{d} ± 0.89
	5	10	3.10 ± 1.97	0.15 ± 0.34	0.15 ± 0.24	0.45 ^{b} ± 0.50	0.85 ± 0.82	1.75 ^{cd} ± 1.45	0.65 ^{abc} ± 0.78	0.80 ^{abc} ± 0.54
	6	5	2.10 ± 2.04	0.00 ± 0.00	0.20 ± 0.27	0.20 ^{ab} ± 0.27	0.20 ± 0.45	1.50 ^{bcd} ± 1.87	0.90 ^{bc} ± 1.34	0.20 ^{ab} ± 0.27
	7	10	1.10 ± 1.15	0.10 ± 0.32	0.00 ± 0.00	0.20 ^{ab} ±0.26	0.10 ± 0.21	0.85 ^{abc} ± 1.11	0.70 ^{abc} ± 0.97	0.10 ^{ab} ± 0.21
	8	10	0.95 ± 0.93	0.00 ± 0.00	0.00 ± 0.00	0.05 ^{a} ± 0.16	0.35 ± 0.24	0.40 ^{ab} ± 0.70	0.10 ^{a} ± 0.21	0.05 ^{a} ± 0.16
	9	10	1.40 ± 1.66	0.00 ± 0.00	0.00 ± 0.00	0.25 ^{ab} ± 0.26	0.35 ± 0.53	0.80 ^{abc} ± 0.86	0.10 ^{a} ± 0.32	1.10 ^{abcd} ± 1.77
	10	10	3.80 ± 2.75	0.05 ± 0.16	0.15 ± 0.47	0.20 ^{ab} ± 0.26	0.35 ± 0.63	1.65 ^{cd} ± 0.85	0.25 ^{ab} ± 0.42	1.30 ^{bcd} ± 1.27
**Total**		**110**	**2.99 ± 5.11**	**0.03 ± 0.16**	**0.27 ± 0.76**	**0.15 ± 0.28**	**0.46 ± 1.41**	**0.92 ± 1.12**	**0.32 ± 0.61**	**0.70 ± 1.14**
**p-value***			0.157	0.537	0.126	0.012	0.55	<0.001	0.003	<0.001

* Significance according GLM test. Different superindexes for each date indicate significant differences according to Duncan's post hoc test (p < 0.05).

Table S4. Mean ± standard deviation of the catches of non-target fauna in Stomoxycc® traps by trap in The Donkey Sanctuary (Western Spain).

Trap	n	Brachycera	Bombyliidae	Apinae	Culicidae	Lepidoptera	Other Diptera	Other Hymenoptera	Syrphidae
4	10	6.30 [b] ± 10.09	0.09 ± 0.25	0.20 ± 0.45	0.18 [ab] ± 0.33	0.95 ± 2.97	1.07 ± 1.27	0.39 ± 0.53	0.27 ± 0.53
8	10	1.41 [a] ± 1.64	0.00 ± 0.00	0.02 ± 0.11	0.30 [b] ± 0.40	0.43 ± 0.62	0.80 ± 0.98	0.23 ± 0.43	0.95 ± 1.55
13	10	2.14 [a] ± 2.55	0.05 ± 0.21	0.50 ± 0.91	0.07 [a] ± 0.18	0.25 ± 0.37	0.55 ± 0.84	0.14 ± 0.38	0.57 ± 0.79
20	5	2.50 [a] ± 1.91	0.00 ± 0.00	0.20 ± 0.57	0.09 [a] ± 0.20	0.30 ± 0.59	1.05 ± 0.91	0.48 ± 0.78	0.80 ± 1.26
21	5	2.61 [a] ± 2.43	0.02 ± 0.11	0.43 ± 1.20	0.11 [a] ± 0.21	0.36 ± 0.58	1.16 ± 1.47	0.36 ± 0.80	0.93 ± 1.22
**Total**	**110**	**2.99 ± 5.11**	**0.03 ± 0.16**	**0.27 ± 0.76**	**0.15 ± 0.28**	**0.46 ± 1.41**	**0.92 ± 1.12**	**0.32 ± 0.61**	**0.70 ± 1.14**
**p-value***		0.011	0.270	0.195	0.030	0.478	0.198	0.276	0.853

* Significance according GLM test. Different superindexes for each trap indicate significant differences according to Duncan's post hoc test ($p < 0.05$).

Table S5. Somer's d correlation test between the five defensive movements of equids against bites of *Stomoxys calcitrans* in The Donkey Sanctuary (Western Spain).

	Score head/neck	Score front legs	Score back legs	Score tail
**Score ears**	0.446 (p<0.001)	0.174 (p=0.003)	0.152 (p=0.008)	0.529 (p<0.001)
**Score head/neck**		0.198 (p=0.004)	0.211 (p=0.002)	0.498 (p<0.001)
**Score front legs**			0.304 (p=0.002)	0.236 (p<0.001)
**Score back legs**				0.224 (p<0.001)

The value is the symmetric Somers' d coefficient and its significance between brackets.

Table S6. Indicator of lesions in donkeys (n = 141) caused by *Stomoxys calcitrans* bites according to the anatomical part over time in the Donkey Sanctuary (Western Spain). Percentage is calculated as the number of animals affected divided into the total, and 95% confidence interval is calculated using the Wilson's Score method. For the period May 2021 to November 2023.

Year	Month	Legs	Face	Chest	Any
2021	5	3.5% (1.5%, 8%)	1.4% (0.4%, 5%)	0.7% (0.1%, 3.9%)	4.3% (2%, 9%)
	6	3.5% (1.5%, 8%)	1.4% (0.4%, 5%)	0.7% (0.1%, 3.9%)	4.3% (2%, 9%)
	7	4.3% (2%, 9%)	1.4% (0.4%, 5%)	1.4% (0.4%, 5%)	5% (2.4%, 9.9%)
	8	4.3% (2%, 9%)	1.4% (0.4%, 5%)	1.4% (0.4%, 5%)	5% (2.4%, 9.9%)
	9	3.5% (1.5%, 8%)	1.4% (0.4%, 5%)	0.7% (0.1%, 3.9%)	4.3% (2%, 9%)
	10	3.5% (1.5%, 8%)	1.4% (0.4%, 5%)	0% (0%, 2.7%)	4.3% (2%, 9%)
	11	3.5% (1.5%, 8%)	1.4% (0.4%, 5%)	0% (0%, 2.7%)	4.3% (2%, 9%)
	12	2.1% (0.7%, 6.1%)	0.7% (0.1%, 3.9%)	0% (0%, 2.7%)	2.8% (1.1%, 7.1%)
2022	1	1.4% (0.4%, 5%)	0.7% (0.1%, 3.9%)	0% (0%, 2.7%)	2.1% (0.7%, 6.1%)
	2	1.4% (0.4%, 5%)	0.7% (0.1%, 3.9%)	0% (0%, 2.7%)	2.1% (0.7%, 6.1%)
	3	2.8% (1.1%, 7.1%)	1.4% (0.4%, 5%)	0% (0%, 2.7%)	4.3% (2%, 9%)
	4	2.8% (1.1%, 7.1%)	0.7% (0.1%, 3.9%)	0% (0%, 2.7%)	3.5% (1.5%, 8%)
	5	2.8% (1.1%, 7.1%)	1.4% (0.4%, 5%)	0.7% (0.1%, 3.9%)	4.3% (2%, 9%)
	6	3.5% (1.5%, 8%)	1.4% (0.4%, 5%)	1.4% (0.4%, 5%)	5% (2.4%, 9.9%)
	7	3.5% (1.5%, 8%)	2.8% (1.1%, 7.1%)	2.8% (1.1%, 7.1%)	4.3% (2%, 9%)
	8	3.5% (1.5%, 8%)	2.8% (1.1%, 7.1%)	2.1% (0.7%, 6.1%)	4.3% (2%, 9%)
	9	3.5% (1.5%, 8%)	2.1% (0.7%, 6.1%)	2.1% (0.7%, 6.1%)	4.3% (2%, 9%)
	10	3.5% (1.5%, 8%)	1.4% (0.4%, 5%)	2.1% (0.7%, 6.1%)	3.5% (1.5%, 8%)
	11	3.5% (1.5%, 8%)	0% (0%, 2.7%)	0% (0%, 2.7%)	3.5% (1.5%, 8%)
	12	5% (2.4%, 9.9%)	2.1% (0.7%, 6.1%)	1.4% (0.4%, 5%)	5.7% (2.9%, 10.8%)
2023	1	5% (2.4%, 9.9%)	2.1% (0.7%, 6.1%)	1.4% (0.4%, 5%)	5.7% (2.9%, 10.8%)
	2	5% (2.4%, 9.9%)	1.4% (0.4%, 5%)	0% (0%, 2.7%)	6.4% (3.4%, 11.7%)
	3	5% (2.4%, 9.9%)	1.4% (0.4%, 5%)	0% (0%, 2.7%)	6.4% (3.4%, 11.7%)
	4	5% (2.4%, 9.9%)	1.4% (0.4%, 5%)	0% (0%, 2.7%)	6.4% (3.4%, 11.7%)
	5	5.7% (2.9%, 10.8%)	2.1% (0.7%, 6.1%)	0% (0%, 2.7%)	6.4% (3.4%, 11.7%)
	6	5.7% (2.9%, 10.8%)	2.8% (1.1%, 7.1%)	0% (0%, 2.7%)	6.4% (3.4%, 11.7%)
	7	5.7% (2.9%, 10.8%)	2.8% (1.1%, 7.1%)	0% (0%, 2.7%)	6.4% (3.4%, 11.7%)
	8	4.3% (2%, 9%)	1.4% (0.4%, 5%)	0% (0%, 2.7%)	5% (2.4%, 9.9%)
	9	4.3% (2%, 9%)	1.4% (0.4%, 5%)	0% (0%, 2.7%)	5% (2.4%, 9.9%)
	10	5% (2.4%, 9.9%)	1.4% (0.4%, 5%)	0% (0%, 2.7%)	5.7% (2.9%, 10.8%)
	11	6.4% (3.4%, 11.7%)	2.1% (0.7%, 6.1%)	0% (0%, 2.7%)	7.1% (3.9%, 12.6%)

Figure S1A. Maximum annual temperatures recorded from the meteorological station in Jerez de Los Caballeros, located at 19 km longitudinal distance from The Donkey Sanctuary (Western Spain) for the period 2021-2023.

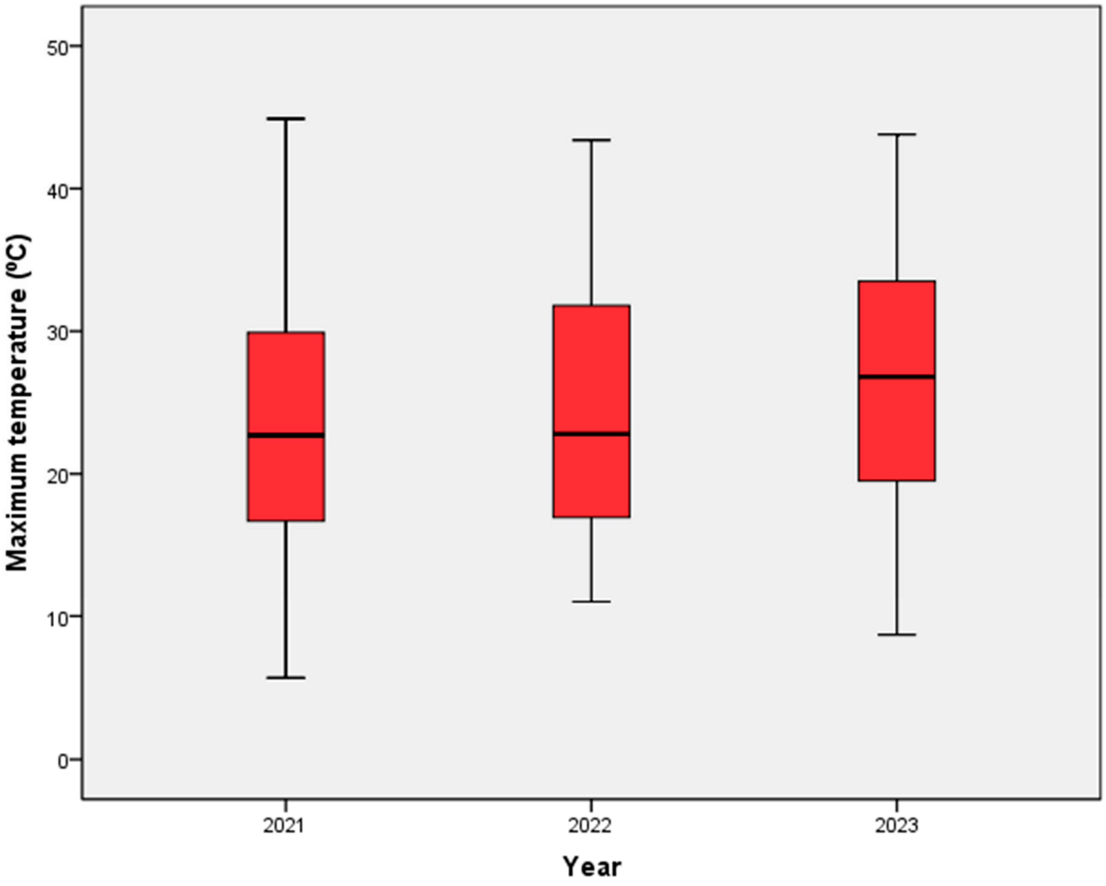

Figure S1B. Maximum monthly temperatures recorded from the meteorological station in Jerez de Los Caballeros, located at 19 km longitudinal distance from The Donkey Sanctuary (Western Spain) for the period 2021-2023.

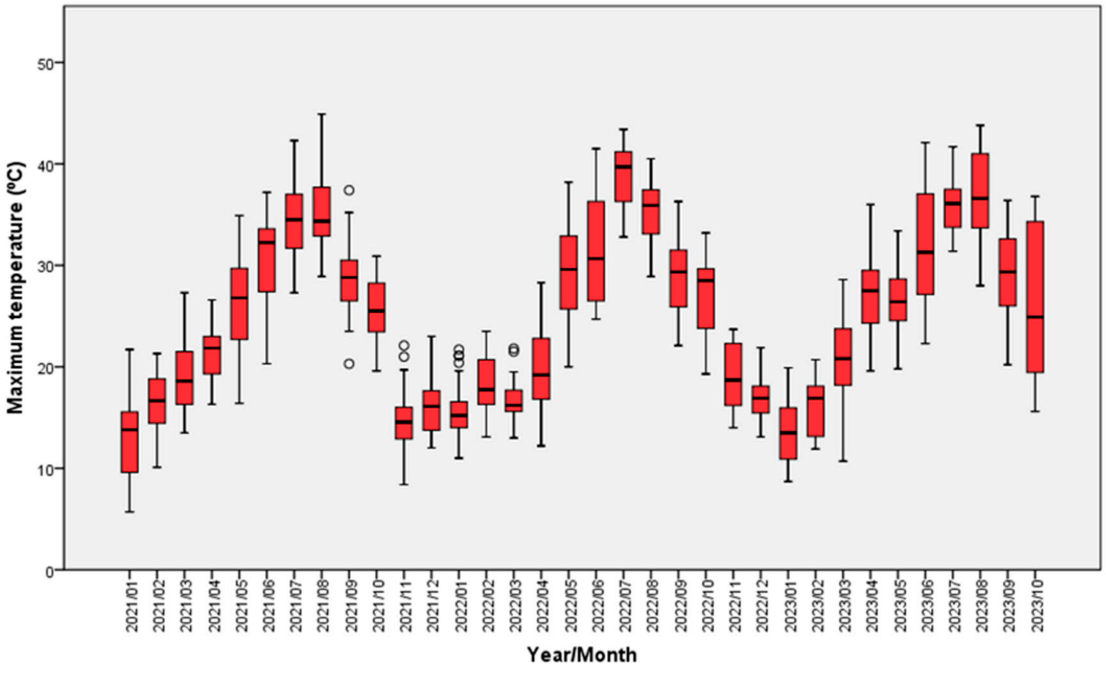

Figure S2A. Minimum annual temperatures recorded from the meteorological station in Jerez de Los Caballeros, located at 19 km longitudinal distance from The Donkey Sanctuary (Western Spain) for the period 2021-2023.

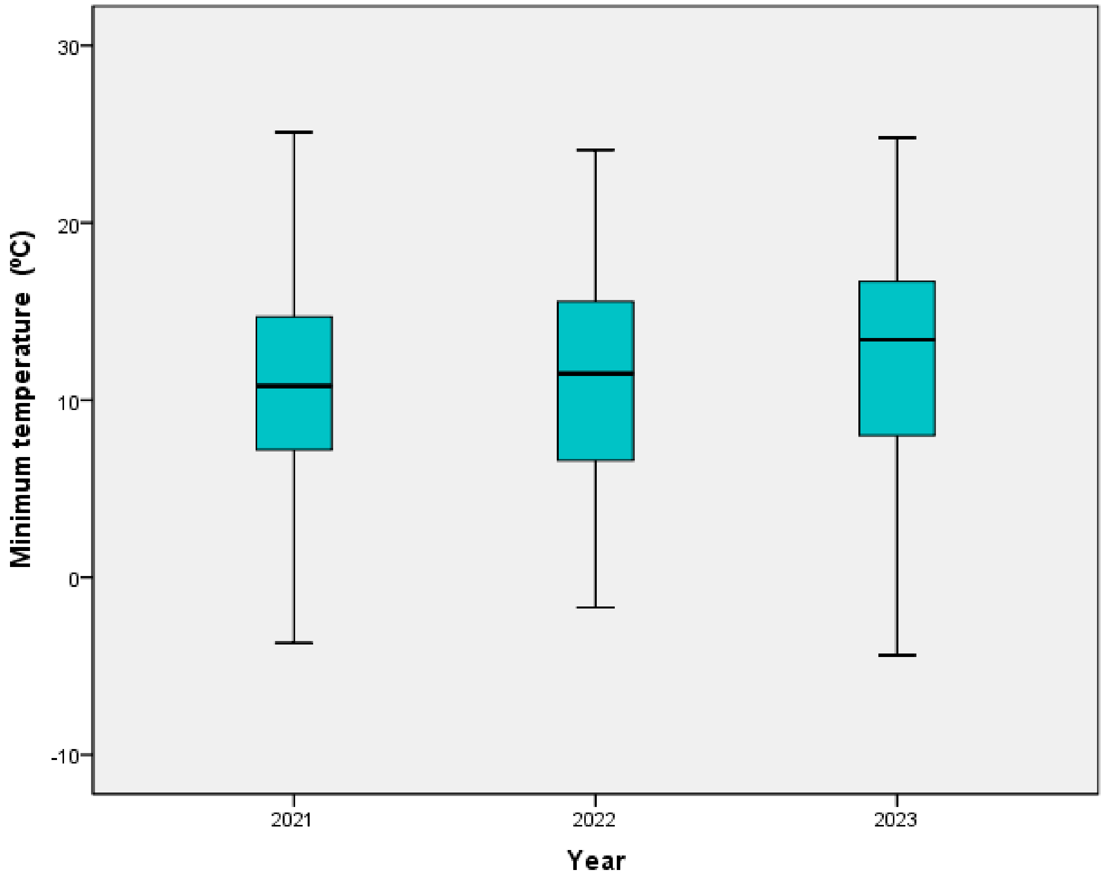

Figure S2B. Minimum monthly temperatures recorded from the meteorological station in Jerez de Los Caballeros, located at 19 km longitudinal distance from The Donkey Sanctuary (Western Spain) for the period 2021-2023.

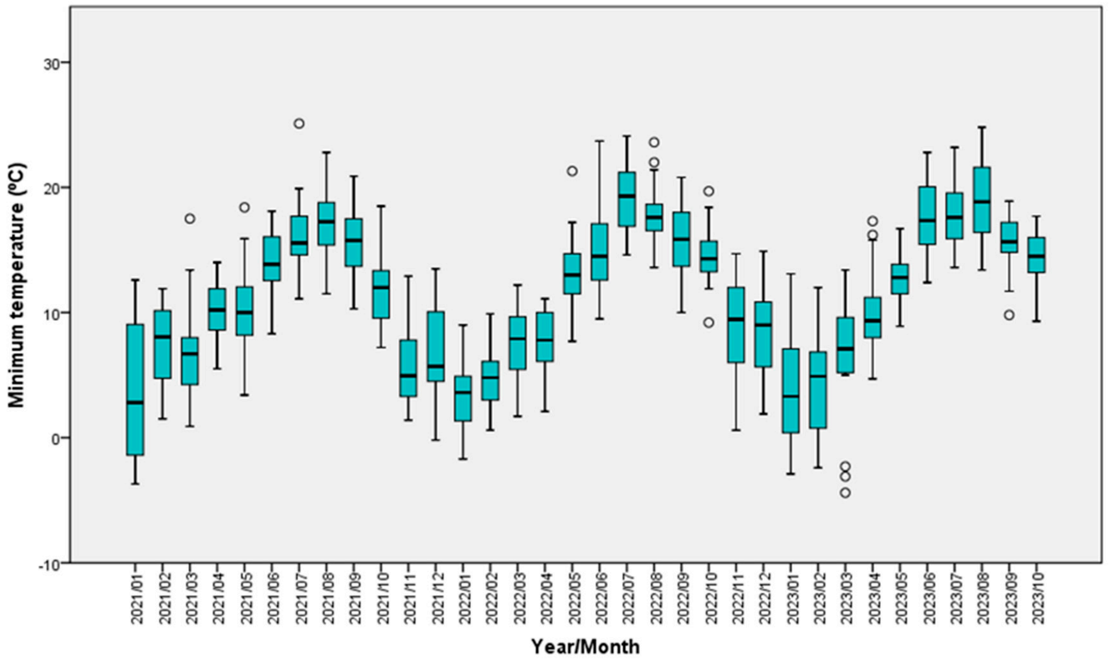

Figure S3A. Annual recorded precipitation from the meteorological station in Jerez de Los Caballeros, located at 19 km longitudinal distance from The Donkey Sanctuary (Western Spain) for the period 2021-2023.

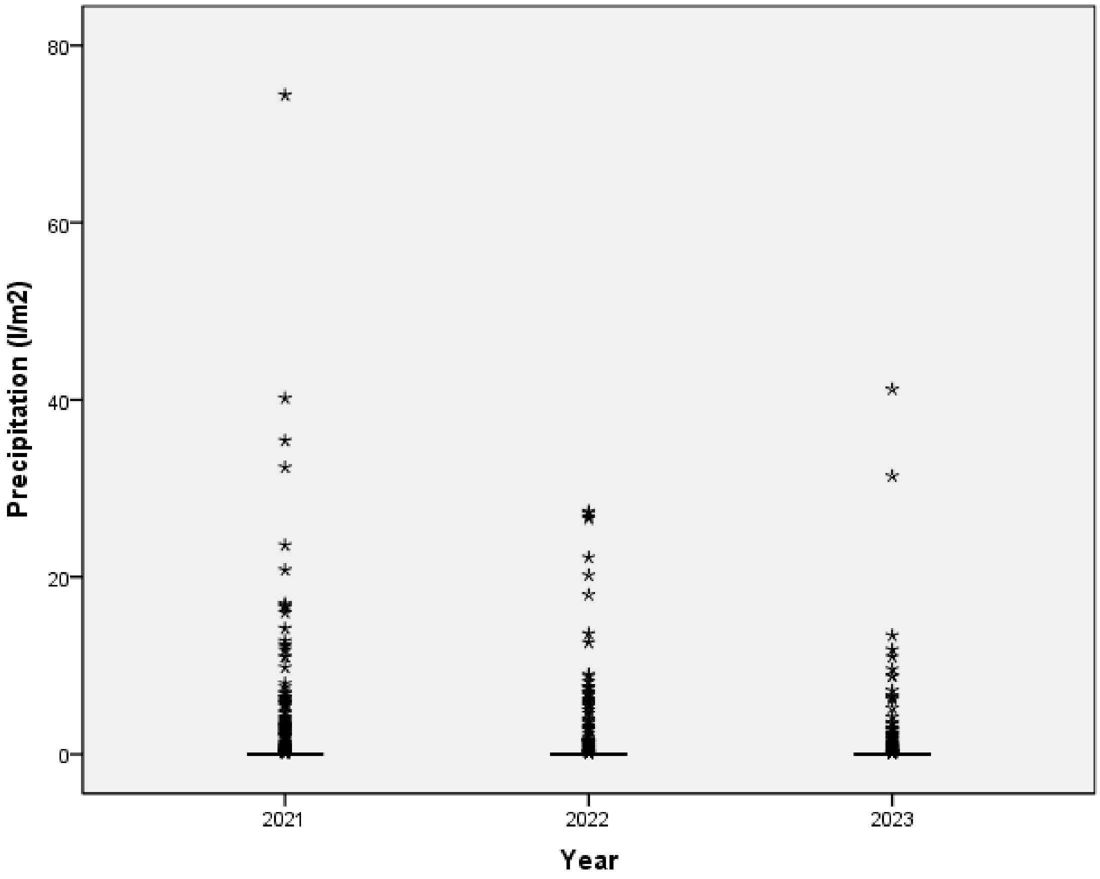

Figure S3B. Monthly recorded precipitation from the meteorological station in Jerez de Los Caballeros, located at 19 km longitudinal distance from The Donkey Sanctuary (Western Spain) for the period 2021-2023.

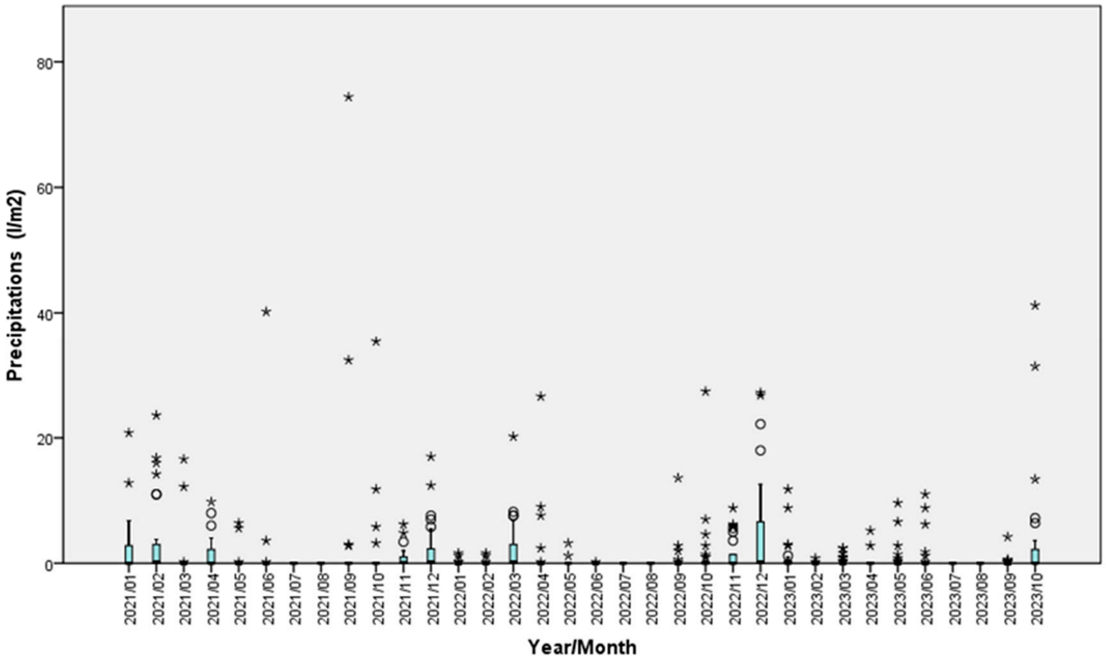

Figure S4A. Daylight hours recorded annually from the meteorological station in Jerez de Los Caballeros, located at 19 km longitudinal distance from The Donkey Sanctuary (Western Spain) for the period 2021-2023.

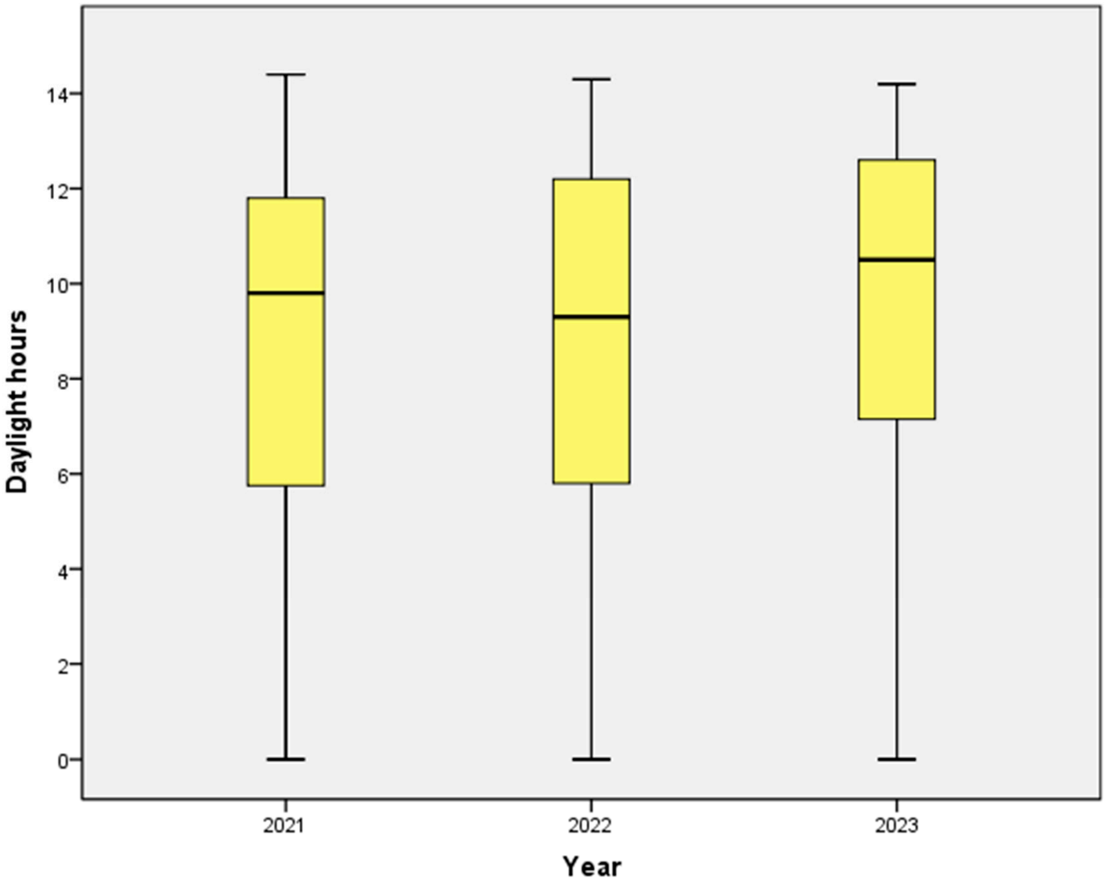

Figure S4B. Daylight hours recorded monthly from the meteorological station in Jerez de Los Caballeros, located at 19 km longitudinal distance from The Donkey Sanctuary (Western Spain) for the period 2021-2023.

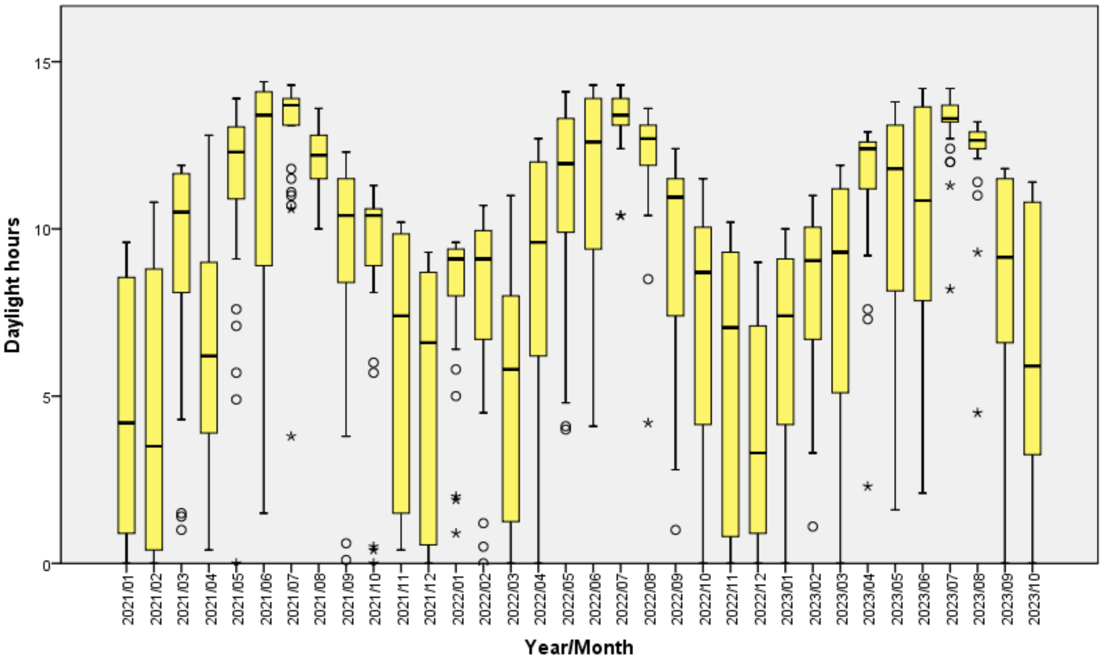

Figure S5. Correlation between extension and severity of skin lesions in donkeys caused by *Stomoxys calcitrans* bites in The Donkey Sanctuary (Western Spain).

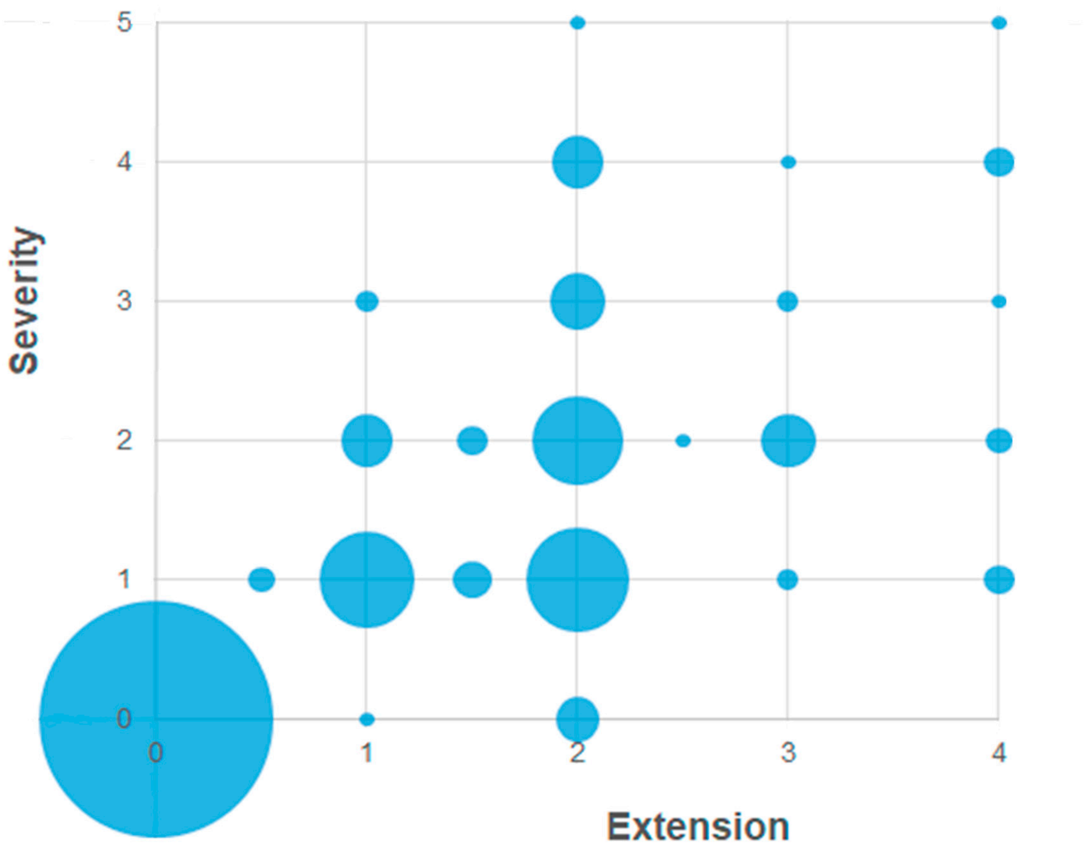

Area of bubbles represents the number of cases.
